# *In vitro *effects of selenium deficiency on West Nile virus replication and cytopathogenicity

**DOI:** 10.1186/1743-422X-5-66

**Published:** 2008-05-31

**Authors:** Saguna Verma, Yanira Molina, Yeung Y Lo, Bruce Cropp, Cheynie Nakano, Richard Yanagihara, Vivek R Nerurkar

**Affiliations:** 1 Retrovirology Research Laboratory, Department of Tropical Medicine, Medical Microbiology and Pharmacology, John A. Burns School of Medicine, University of Hawaii at Manoa, Honolulu, HI 96813, USA; 2 Department of Pediatrics, John A. Burns School of Medicine, University of Hawaii at Manoa, Honolulu, HI 96813, USA; 3 Asia-Pacific Institute of Tropical Medicine and Infectious Diseases, John A. Burns School of Medicine, University of Hawaii at Manoa, Honolulu, HI 96813, USA

## Abstract

**Background:**

Selenium (Se) deficiency plays an important role in viral pathogenesis. To understand the effects of Se deficiency on West Nile virus (WNV) infection, we analyzed cytopathogenicity, apoptosis and viral replication kinetics, using a newly developed Se-deficient cell culture system.

**Results:**

Both Vero and SK-N-SH cells grown in Se-deficient media exhibited a gradual loss of glutathione peroxidase (GPx1) activity without any significant effect on cell growth and viability. In SK-N-SH cells, Se deficiency had no effect on the expression of key antioxidant enzymes, including manganese- and copper-zinc superoxide dismutase (MnSOD and CuZnSOD), catalase and inducible nitric oxide synthase, whereas Vero cells demonstrated a significant increase in the expression of MnSOD and an overall increase in oxidative stress (OS) at day 7 post-induction of Se deficiency. At 2 days after infection with WNV, CPE and cell death were significantly higher in WNV-infected Se-deficient Vero cells, compared to WNV-infected control cells. Furthermore, WNV-induced apoptosis was significantly heightened in Se-deficient cells and was contributed by loss of mitochondrial membrane potential and increased caspase activity. However, no significant difference was found in WNV copy numbers between control, Se-adequate and Se-deficient cell cultures.

**Conclusion:**

Overall results demonstrate that the *in vitro *Se-deficient model can be used to study responses of WNV to this essential nutrient. Although Se deficiency has no *in vitro *effect on WNV replication kinetics, adequate Se is presumably critical to protect WNV-infected cells against virus-induced cell death.

## Background

Selenium (Se), an essential trace mineral, contributes significantly to host immune responses and antioxidant protection, due to its incorporation as selenocysteine in glutathione peroxidases (GPx) [1]. As such, impaired antioxidative and immune responses associated with inadequate dietary Se results in increased disease severity following infections with HIV, influenza virus and Coxsackie virus [2,3]. In HIV- infected patients, low plasma Se levels are associated with the development of severe cardiomyopathy [4,5]. Similarly, experimental and epidemiologic studies indicate that low dietary Se increases the risk of hepatocellular carcinoma in carriers of hepatitis B and C viruses [6]. Moreover, point mutations in Coxsackie virus B3 (CVB3/0) and influenza A virus (H3N2) have been associated with increased disease severity in Se-deficient mice [7-9], and an increase in reactive oxygen species (ROS) was demonstrated to enhance HIV replication in T-lymphocytic and monocytic cells [10-12]. Thus, Se deficiency leads to increased virulence and evolution of viral quasispecies [13,14].

West Nile virus (WNV), a mosquito-borne flavivirus which causes lethal encephalitis in humans and horses, is maintained in an enzootic cycle between many mosquito and bird species [15-18]. The unexpected emergence of WNV in the United States in 1999 was associated with the introduction of the NY99 strain which is more virulent, replicates more efficiently with severe cytopathogenic effects (CPE), and results in higher incidences of meningoencephalitis in humans as compared to the avirulent Eg101 strain [15,16].

While Se deficiency is known to influence oxidative stress (OS) and host immune responses, the specific mechanism(s) driving the severity of host pathology as well as viral mutations remains largely unknown. Most studies to date have focused on *in vivo *experiments using animals fed Se-deficient diets and the complexity of *in vivo *experiments does not allow a full understanding of the precise cellular and molecular mechanisms responsible for virus mutations, selection and enhanced pathogenesis. Establishment of tissue-culture systems of Se deficiency-induced OS response will allow a more detailed analysis of the molecular mechanisms associated with nutritional deficiency of Se as an antioxidant and its role in the emergence of quasispecies with heightened disease potential. Data on the induction of Se deficiency in an *in vitro *cell-culture system is limited and suggest a cell-specific response [19-21]. Cells, such as Jurkat E6-1 (human T-leukemic) cells, undergo rapid apoptotic cell death within 24 hr after Se supplementation, whereas murine macrophage cells (RAW.21) survive for 8–12 passages in a Se-deficient state [20,22]. To delineate the specific effect of dietary Se on virus infection, it is important to identify cell lines in which Se deficiency can be efficiently induced *in vitro *without compromising cell viability.

Based on the Se-deficient *in vitro *and *in vivo *pathogenesis studies using HIV, H3N2 and CVB3/0 [2,23], we hypothesized that OS induced by Se deficiency may play an important role in WNV pathogenesis. As a first step towards associating the role of Se deficiency in WNV pathogenesis, we developed an *in vitro *Se-deficient model using Vero cells, which efficiently supports WNV infection, and human neuronal cells (SK-N-SH), the natural target of WNV in the brain. Furthermore, we infected Se-deficient Vero cells with WNV and compared the WNV replication kinetics, cytopathogenicity and virus-induced apoptosis with cells grown with Se-adequate media. Our data demonstrate that Se deficiency can be induced in Vero and SK-N-SH cells, and WNV infection of Se-deficient Vero cells leads to enhanced cell death by apoptosis and CPE without altering WNV replication kinetics.

## Results

### Effect of Se deficiency on Vero and SK-N-SH cells

**Figure 1:**
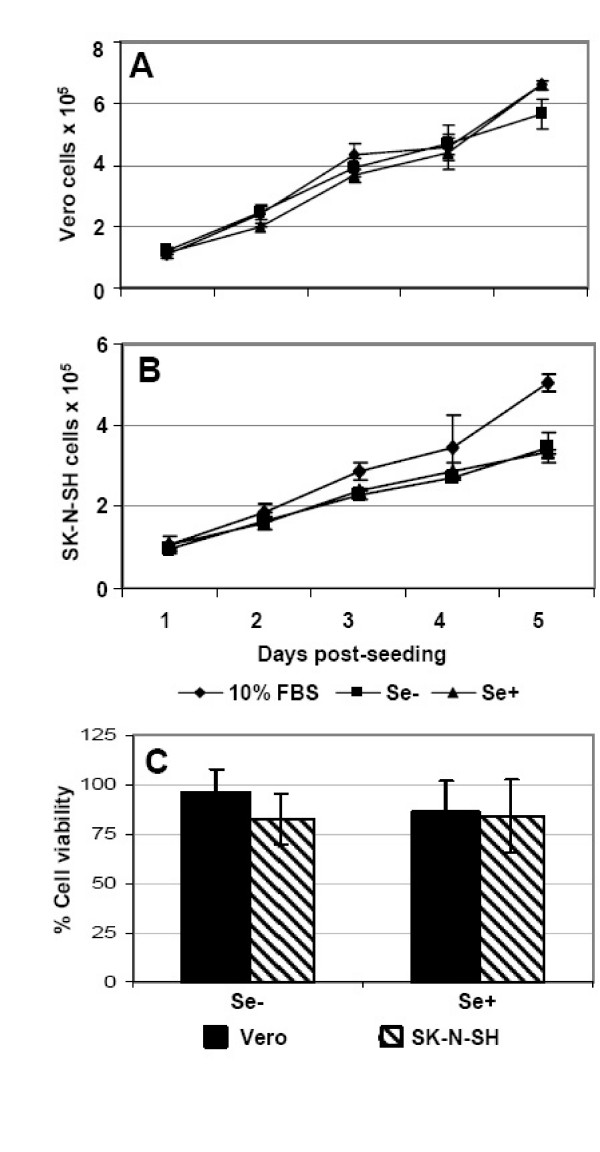
**In vitro response of Vero and SK-N-SH cells to Se deficiency**. Vero and SK-N-SH cells were grown in Se-deficient (Se-) and Se-adequate (Se+) conditions as described in the materials and methods. Equal number of cells were seeded in 96-well plates and growth curve was measured by cell counting of control, Se-, and Se+, Vero **(A) **and SK-N-SH **(B) **cells for 5 days post-seeding. **(C) **Cell viability of Vero and SK-N-SH cells at day 7 of the induction of Se deficiency was assessed by cell proliferation assay and percentage cell viability of Se- and Se+ cells was calculated by comparing to control cells. Data are expressed as mean ± SD from two separate experiments performed in triplicate.

FBS is the main source of Se for cells grown *in vitro*. Thus, low Se levels were achieved by reducing the FBS concentration from 10% to 1%. Since lowering the FBS concentration reduces the essential growth factors in the media, we supplemented the media with insulin and transferrin and changed the media every two or three days to maintain cell growth and proliferation. Exogenous Se added in Se deficient medium was used as a positive control in all experiments to confirm the specificity of Se in the oxidant/antioxidant response and cytopathogenicity induced by WNV. Growth rates of Vero and SK-N-SH cells, as measured by cell counting, were not affected by reducing FBS from 10% to 1% (Fig. 1A and 1B). However, SK-N-SH cells upon confluence displayed slightly slower growth on day 4 and 5 and therefore were passaged on day 4 post-seeding to maintain comparable growth patterns in all the treatments. Further, cell viability of Vero and SK-N-SH cells was measured at day 3 and day 7 post-induction of Se deficiency. At day 3, there was no change in the cell viability of Se-deficient and Se-adequate cells as compared to control cells with 10% FBS (data not shown). At day 7, the cell viability of Se-deficient and Se-adequate Vero and SK-N-SH cells was between 80–100% as compared to cells grown in control media, which was statistically not significant (Fig. 1C). These results indicate that the medium containing 1% FBS, insulin and transferrin was adequate for growth of Vero and SK-N-SH cells for 10–12 days.

**Figure 2:**
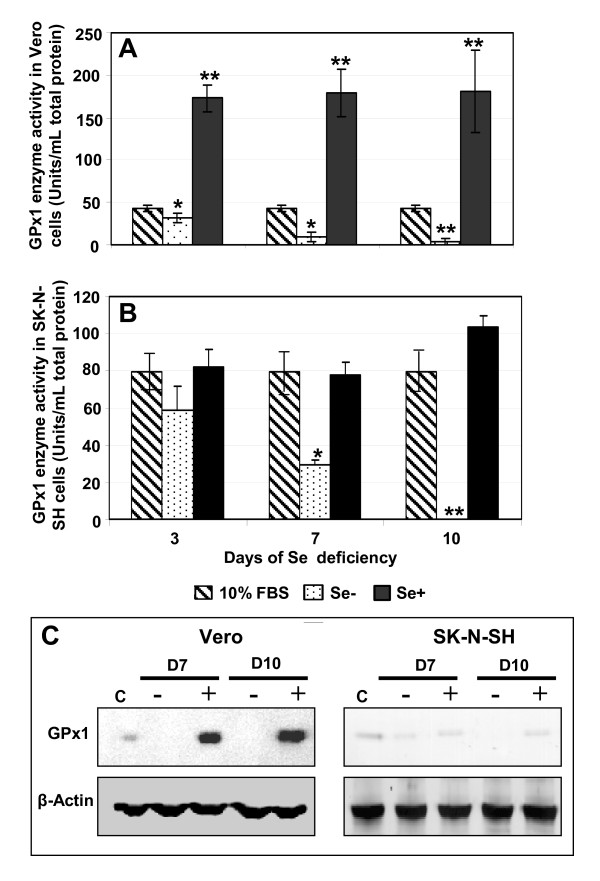
**Effects of Se deficiency on GPx1 enzyme**. *In vitro *Se deficiency was tested by loss of GPx1 enzyme activity. Total soluble proteins were extracted from control, Se- and Se+ Vero **(A) **and SK-N-SH **(B) **cells and GPx1 enzyme activity was measured at days 3, 7 and 10 post-induction of Se deficiency by using the cGPx1 assay kit. Data are reported as mean ± SD of triplicate experiments. * p < 0.05, ** p < 0.005 compared to control cells. **(C) **Analyses of GPx1 protein by Western blot. 50 μg of total protein extracted from Vero and SK-N-SH cells grown in control, Se- and Se+ media were separated on PAGE, followed by immunoblotting with anti-GPx1. Equal loading was confirmed by re-blotting the same membranes with anti-β-actin.

Se-deficient cells were maintained for 10 days and passaged every 3 days using serum-free trypsin-EDTA solution and the GPx1 enzyme activity was measured at days 3, 7 and 10 post-induction of Se deficiency. In both cell types, the loss of GPx1 enzyme activity in Se-deficient cells was significant; however, the enzyme kinetics was different. Vero cells showed a slight decline in GPx1 enzyme activity at day 3, which became significantly lower at day 7 and 10 post-induction of Se deficiency (Fig. 2A). Moreover, exogenous addition of Se in the form of sodium selenite (50 mM) significantly induced GPx1 enzyme activity, almost three times of the control cells, by day 3 and the enzyme levels were consistently high until day 10 (Fig. 2A). Interestingly, the basal activity levels of GPx1 in SK-N-SH control cells were much higher than that in Vero control cells (80 vs. 44 units/mg protein). As expected, Se depletion resulted in a rapid decline of GPx1 activity, starting at day 3 and enzyme activity was undetectable on day 10 (Fig. 2B). However, in contrast to Vero cells, the addition of exogenous sodium selenite did not induce GPx1 enzyme activity, but normalized it to control levels in SK-N-SH cells (Fig. 2B).

Similarly, GPx-1 protein analysis by Western blot confirmed loss of GPx-1 protein at all time points in both cell types (Fig. 2C). Addition of sodium selenite significantly induced GPx1 protein levels in Vero cells but not in SK-N-SH cells, thus supporting the enzyme activity data. Overall, GPx1 enzyme activity and protein expression data indicated that a Se-deficient state was achieved in both the Vero and SK-N-SH cell lines.

### Effects of Se deficiency on antioxidant enzymes

**Figure 3:**
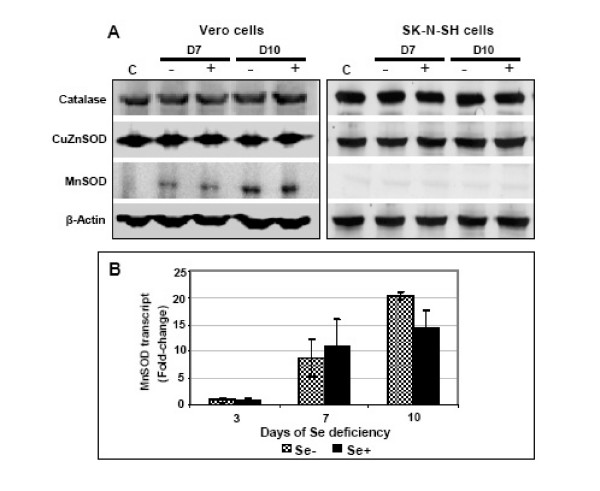
**Effects of Se status on antioxidant enzymes**. **(A) **50 μg of cellular proteins extracted from Vero and SK-N-SH cells grown in control, Se- and Se+ media at days 7 and 10 post-induction of Se deficiency were separated on PAGE, transferred onto nitrocellulose membranes and immunoblotted with antibodies specific to catalase, CuZnSOD and MnSOD. Equal loading of protein was validated by re-blotting the same membranes with anti-β-actin. The data is representative of three independent experiments. **(B) **Increase in the expression of MnSOD in Se- and Se+ Vero cells was confirmed by qRT-PCR. cDNA template was synthesized from total RNA extracted from control, Se- and Se+ Vero cells at days 3, 7 and 10 post-induction of Se deficiency as described in the materials and methods and subjected to qRT-PCR using primers specific for MnSOD and β-actin. Changes in the levels of MnSOD transcripts in Se- and Se+ Vero cells were first normalized to β-actin and then the fold-change as compared to controls was calculated. Data are reported as mean ± SD of triplicate experiments.

Total cellular protein was extracted from control, Se-deficient and Se-adequate Vero and SK-N-SH cells and the profile of antioxidant enzymes, such as CuZnSOD, MnSOD, catalase and iNOS, were characterized by Western blotting at days 7 and 10 post-induction of Se deficiency. As shown in Fig. 3A, induction of Se deficiency had no effect on catalase and CuZnSOD protein levels, while MnSOD protein expression was significantly induced in both Se-deficient and Se-adequate Vero cells. On the other hand, SK-N-SH cells did not show any change in the protein levels of all three antioxidant enzymes (Fig. 3A). iNOS was undetectable in normal, Se-deficient and Se-adequate Vero and SK-N-SH cells at all time points (data not shown). To further verify and quantitate the induction of MnSOD in Vero and SK-N-SH cells, we analyzed the mRNA expression of MnSOD using qRT-PCR (Fig. 3B). Although, our data did not indicate any change in MnSOD transcripts at day 3 post-induction of Se deficiency, an 8- to 20-fold increase in the MnSOD transcripts were observed at days 7 and 10 post-induction of Se deficiency in Vero cells. However, in SK-N-SH cells there was no increase in MnSOD transcripts at all time points (data not shown), further confirming our Western blot data (Fig. 3A).

### Se deficiency increases OS in Vero cells

**Figure 4:**
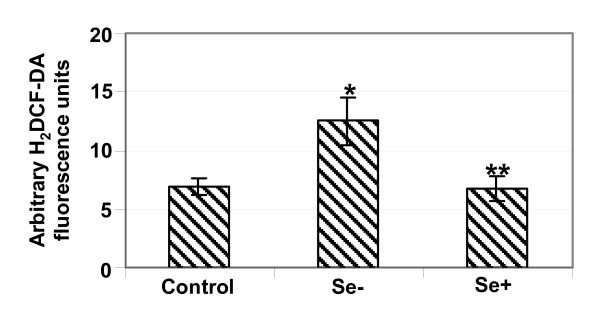
**Se deficiency increases cellular oxidative status**. Vero cells grown in control, Se- and Se+ media in 96-well plates were incubated with 5 μM of 2',7' dichlorodihydrofluorescein diacetate (H_2_DCF-DA) for 30 min, washed twice with PBS and the cell fluorescence, an indicator of overall OS, was read at 485 Ex/535 Em. Arbitrary fluorescence units for each sample, representative of mean ± SD of two independent experiments performed in triplicate are given. *p < 0.05 and **p < 0.05 as compared to control and Se- cells, respectively.

Se is an integral part of the active site of GPx1, an enzyme that protects cell damage by reducing intracellular H_2_O_2 _to water and oxygen. Diminished level of GPx1 results in the accumulation of H_2_O_2_, which was assayed using H_2_DCF-DA, a cell-permeable indicator of intracellular ROS. Incubation of Vero cells with H_2_DCF-DA indicated that Se-deficient cells were under OS (Fig. 4). Addition of exogenous sodium selenite further protected the cells from OS as indicated by a significant decrease of H_2_DCF-DA fluorescence (Fig. 4). Our data demonstrate that OS can be induced in Vero cells using the aforementioned culture conditions and these cells can be effectively used to study the effect of Se on viral infection.

### Se deficiency increases apoptosis in WNV-infected Vero cells

**Figure 5:**
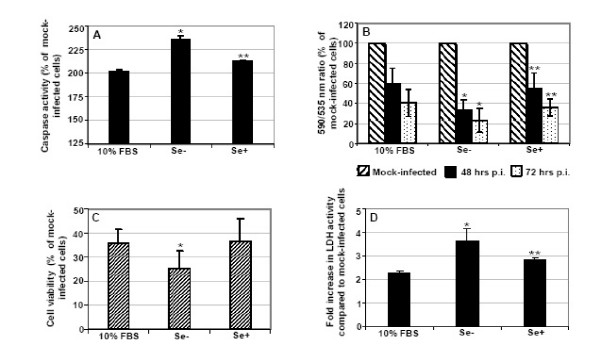
**Effects of Se deficiency on WNV-induced apoptosis and cell death**. Vero cells grown in control, Se-, and Se+ media were infected with WNV at MOI 1 for 2 hr at day 7 post-induction of Se deficiency. After adsorbtion, the cells were washed and maintained in control M199 medium, Se- and Se+ media. **(A) **Caspase 3/7 activity was analyzed using fluorogenic substrate at day 2 after infection in WNV-infected and mock-infected cells. The data are expressed as percentage increase of caspase activity in infected cells grown in control, Se-, and Se+ media as compared to corresponding mock-infected cells. *p < 0.05 and **p < 0.05 as compared to cells grown in control and Se- media, respectively. **(B) **loss of mitochondrial membrane potential is represented as ratio of fluorescence at 590 and 535 nm measured by JC-1 staining at 48 and 72 hr after infection, and expressed as percentage decline in infected cells grown in control, Se-, and Se+ media as compared to corresponding mock-infected cells. *p < 0.05 and **p < 0.05 as compared to cells grown in control and Se- media, respectively. **(C) **Cell viability of infected and mock-infected Vero cells at day 2 after infection was assessed by cell proliferation assay and percentage cell viability of WNV-infected control, Se- and Se+ cells was calculated by comparing to their respective mock-infected cells. *p < 0.05 as compared to WNV-infected Vero cells grown in control media. **(D) **WNV-infected Vero cells grown in control, Se-, and Se+ media were analyzed for LDH levels at day 2 after infection and expressed as fold-change over levels present in mock-infected cells. *p < 0.05 and **p < 0.05 as compared to cells grown in control and Se- media, respectively. All the data are presented as mean ± SD of at least two independent infections performed in triplicate.

Vero cells were inoculated with WNV NY99 strain at multiplicity of infection (MOI) 1, at day 7 post-induction of Se deficiency, to study the kinetics of virus replication and cytotoxicity caused by WNV in control, Se-deficient and Se-adequate cells. WNV infection has been shown to cause apoptotic cell death in Vero cells [24], and activation of caspases play an important role in mediating apoptosis. Therefore, Vero cells grown in control, Se- deficient and Se-adequate media were first subjected to fluorometric assay of caspase-3/7 at day 2 after infection. There was an approximately 200% increase in caspase-3/7 activity in WNV-infected Vero cells grown in control media compared to naïve control cells, which further increased significantly to 240% (p < 0.01) in WNV-infected Se-deficient cells as compared to naïve Se-deficient cells (Fig. 5A). Presence of exogenous Se in the media could partially modulate the increase in the caspase activity. Another hallmark of apoptosis is mitochondrial dysfunction. We therefore analyzed the change in mitochondrial membrane potential (ΔΨm), a marker of mitochondrial dysfunction, using the fluorescent probe JC-1 in infected and mock-infected control, Se-deficient and Se-adequate cells. JC-1 is selectively taken up into the mitochondria and is a reliable indicator of ΔΨm [25]. At hyperpolarized ΔΨm, JC-1 forms J aggregates in a rapidly reversible manner, emitting red fluorescence, while during depolarization of mitochondria, JC-1 leaks and consequently reduces dye content in mitochondrial matrix and emits a green florescence [26]. Ratiometric measurement of red to green JC-1 fluorescence indicates ΔΨm. As seen in Figure 5B, a 40% and 60% loss in the ΔΨm was observed in the WNV-infected control Vero cells at 48 and 72 hr after infection, respectively, as compared to mock-infected control cells. This loss of ΔΨm further decreased significantly to 65% and 80% in WNV-infected Se-deficient cells at the same time points, respectively, as compared to mock-infected Se-deficient cells (p < 0.05). At both the time points, the presence of exogenous Se partially reversed the loss of ΔΨm. The difference between control and Se-adequate cells was not statistically significant in Figure 5A and 5B.

### Se deficiency increases cytopathogenicity of WNV-infected Vero cells

The WNV-induced cytotoxicity in infected cells was detected by measuring the cell viability and LDH levels. Decrease in cell viability and increase in LDH activity has been previously reported in WNV-infected Vero cells between 32 to 48 hr after infection [24]. Also, the percentage cell viability of WNV-infected Vero cells grown in control and Se-adequate media at 48 hr after infection was approximately 35% of their respective naïve Vero cells. However, the cell viability decreased to 25% (p < 0.05) in WNV-infected Se-deficient cells when compared to naïve Se-deficient cells (Fig. 5C). Similarly, LDH activity which was 2.2- and 2.8-fold higher in WNV-infected Vero cells grown in control and Se-adequate media as compared to the respective naïve Vero cells, further increased significantly to 3.6-fold (p < 0.05) in infected Se-deficient cells as compared to naïve Se-deficient cells (Fig. 5D). Phase-contrast microscopy of mock-infected control, Se-deficient and Se-adequate Vero cells at day 3 after infection indicated intact homogenous nuclei and cell boundaries. On day 3 after infection, noticeable CPE, such as rounding of cells, swelling of nuclei and distortion of cell monolayers were observed in control Vero cells, which concurred with previously published data on WNV-induced CPE in Vero cells [24]. However, at the same time point, rounding of cells with enlarged nuclei and distorted cell boundaries were observed in more than 60% of Se-deficient WNV-infected Vero cells, compared to WNV-infected control and Se-adequate cells (data not shown). These results further support the cell-viability data observed in WNV-infected Se-deficient cells.

### Se deficiency has no effect on WNV viral replication

**Figure 6:**
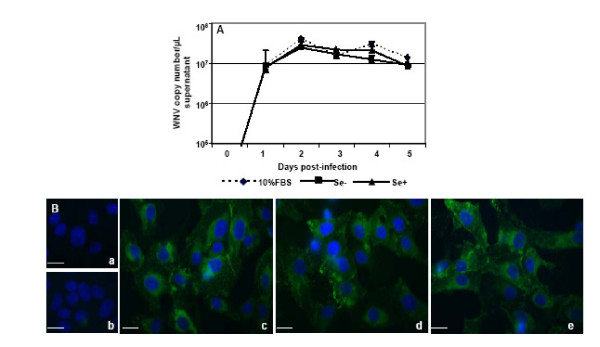
**WNV replication kinetics in Se deficient cells**. **(A) **Vero cells grown in control, Se-, and Se+ media were infected with WNV at day 7 post induction of Se deficiency and cell supernatants were harvested every 24 hr for 5 days. Viral RNA extracted from the cell supernatant was used to determine viral copy number by qRT-PCR and expressed as viral copy number per mL. Data represents mean ± SD of three independent infections. **(B) **WNV-infected control, Se- and Se+ Vero cells grown and fixed on coverslips at day 2 post infection were incubated with monoclonal human anti-WNV env antibody and then with Alexa Fluor 488 conjugated goat anti-mouse secondary antibody. Mock infected Vero cells **(a) **and infected Vero cells stained with secondary antibody alone **(b)**, were used as a negative control. The experiments were performed in triplicate and **c**, **d **and **e **represents WNV antigen staining in Vero cells grown in control, Se- deficient and Se- adequate media, respectively. Scale bar represents 10 μm at a magnification of 63× in all pictures.

To further analyze the effect of Se deficiency on viral replication kinetics, viral copy numbers were determined in the WNV-infected supernatants at different time points after infection. qRT-PCR analysis of the viral RNA extracted from cell supernatants indicated rapid increase in virus replication between 12 to 24 hr after infection, which peaked at day 2 after infection, and continued until day 5 after infection (Fig. 6A). However, WNV copy numbers did not differ between control (10% FBS), Se-deficient and Se-adequate Vero cells. Based on epifluorescence microscopy, approximately 80% of infected Vero cells expressed strong immunoreactivity to WNV envelope antigen at day 2 after infection (Fig. 6B). No staining was observed in mock-infected and WNV-infected Vero cells incubated with only secondary antibody (Fig. 6B, a and 6b). However, there was no difference in the staining pattern between WNV-infected control, Se-deficient and Se-adequate cells, thus supporting our WNV copy number data (Fig. 6B, c, d and 6e).

## Discussion

The role of Se and OS in infectious diseases has been associated with changes in the host immune system and the viral pathogen *per se *[2,27]. The factors that influence severity of WNV-associated pathology are largely unknown. Because high WNV titers in the blood and peripheral tissues are correlated with early virus entry into the central nervous system, it is important to analyze the factors that might influence virus replication, mutations and cytopathogenicity in cells in which the virus replicates. One such factor that may affect virulence and/or cytopathogenicity is Se deficiency-associated OS. This study was therefore initiated based on the hypothesis that Se deficiency-associated OS might influence the replication and cytopathogenicity of WNV. In this report, we describe a Se-deficient *in vitro *culture system using Vero and SK-N-SH cells, in which OS can be induced without significant effect on cell growth and cell viability. Additionally, we demonstrate that WNV infection of Se-deficient Vero cells leads to profound CPE and enhanced virus-induced apoptosis without significantly affecting WNV replication kinetics.

### Development of Se-deficient *in vitro *model

Previous studies on Se mostly involved mouse models to study viral pathogen response, which did not allow differentiating between Se-induced immune and OS responses. Limited data on *in vitro *Se-deficient models predict a very cell-specific response [19,20,22]. Saito and colleagues demonstrated that within 24 hr, Se deficiency significantly decreased GPx1 enzyme activity and induced apoptosis in Jurkat E6 cells [20]. In another study, human hepatoma (Huh) cells displayed morphological changes as a result of apoptotic cell death, at day 4 post-induction of Se deficiency [28]. As expected, Se deficiency decreased GPx1 enzyme activity and increased OS parameters in both studies. Similarly, our study demonstrated a progressive reduction in GPx1 enzyme activity when cells were propagated in 1% FBS, however without significantly affecting cell proliferation rate of Se-deficient cells as compared to Se-adequate and control cells. This may be either because Vero and SK-N-SH cells tolerate Se deficiency better than some of the previously studied cell lines and/or due to the method used to induce Se deficiency in our *in vitro *model. In studies employing Jurkat E6 cells, the Se-deficient medium comprised of insulin, transferrin and bovine serum albumin as a substitute for FBS, whereas in studies employing Huh cells, Se deficiency was induced by growing cells with media containing 0.01% fetal calf serum without any exogenous growth factors [20,28]. It is likely that the cell death seen in both the aforementioned studies is partly because the cells were deprived of vital growth factors and other nutrients, such as vitamins E and C provided by FBS for the normal growth of cells in an *in vitro *system. Our results indicate that lowering the FBS concentration to 1% and supplementing the media with growth factors is sufficient to induce Se deficiency without any significant effect on the viability of Vero and SK-N-SH cells. Similar observations have been noted when Se deficiency was induced without significant effect on cell growth in mouse monocyte-macrophage cells (RAW 264.7) and bovine mammary endothelial cells by growing them for 8–16 passages in 2–5% FBS [22,29].

### Se deficiency, antioxidants enzymes and OS

Comparison of the responses of Vero and SK-N-SH cells to Se deficiency revealed much higher basal levels of GPx1 enzyme activity in SK-N-SH cells than in Vero cells. Though Se deficiency induced loss of GPx1 activity in both cell types, exogenous addition of 50 nM sodium selenite significantly induced GPx1 enzyme activity (3-fold) and MnSOD levels (8- to 20-fold), in Se adequate Vero cells, whereas similar treatment of SK-N-SH cells did not elicit such a robust response. GPx1 enzyme is present in the cytosol and mitochondrial matrix and its prevalence in different tissues varies depending on their metabolic activities and exposure to oxygen [30]. Brain is an organ, which metabolically consumes 20% of the total oxygen, and neurons and the glial cells are reported to harbor high enzymatic activities of antioxidant enzymes. Our results demonstrating higher basal protein levels and activity of GPx1 enzyme in neuron-derived SK-N-SH cells concur with the above observation. However, we were surprised that exogenous sodium selenite did not further induce GPx1 enzyme activity in SK-N-SH cells as seen in Vero cells suggesting different feedback regulatory mechanisms involved in these two cell types.

MnSOD is an important mitochondrial Se-independent antioxidant enzyme and has been reported to be regulated by ROS-induced changes in cellular redox status in several cell types [31,32]. Though direct influence of Se deficiency on MnSOD is not reported in culture system, recently Styblo and colleagues reported increased MnSOD in lung tissue of Se-deficient mice and have related the surge in H_2_O_2_to both, the increased MnSOD activity and loss of GPx1 activity [33]. Further, it has been recently shown by Jaspers and co-workers that primary human bronchial epithelial cells grown in Se-deficient media exhibited significantly lower catalase enzyme activity, whereas there was no change in CuZnSOD enzyme activity [19]. Based on the results of Jaspers and colleagues, and our data demonstrating differential responses of MnSOD expression in Se-deficient Vero and SK-N-SH cells, it appears that the Se-mediated regulation of Se-independent antioxidant enzymes is a cell-specific, rather than a common response to Se deficiency. Literature on the effects of sodium selenite treatment and/or over expression of GPx1 on other antioxidant enzymes is lacking. However the drastic increase in MnSOD levels in Se-adequate Vero cells may be due to the significant induction of GPx1 enzyme in sodium selenite treated Vero cells. Since the transcription of MnSOD is under the control of cellular redox status, both increase in ROS or antioxidant defense enzymes may result in its induction.

### Role of Se in viral pathogenesis

Several lines of laboratory evidence support the pathogenic effects of inadequate Se in viral infections [27,34-37]. Antioxidant nutrient deficiencies have been shown to hasten progression of viral diseases, and both, clinical and *in vitro *studies to assess Se supplementation as an adjuvant therapy for HIV-infected patients are encouraging [36,37]. An inverse correlation of Se status and mortality in HIV-infected patients is linked to the ability of Se to boost cellular and humoral immunity by up-regulating the activity of natural killer and cytotoxic T cells [11,38,39]. Apart from viral mutations, increased viral cardiovirulence and heart damage was observed in CVB3/0-infected mice fed with only Se-deficient diet or coupled with vitamin E-deficient diet [40]. Similar to the *in vivo *studies described above, *in vitro*, Vero cells are routinely used to study flaviviruses such as, DENV, JEV and WNV [24,41,42], and neurons are the targets of several viral and bacterial pathogens [43-46]. However, the role of Se in the pathogenesis of flavivirus-associated diseases has not been explored.

Our results demonstrate that WNV replicates as efficiently in Se-deficient cells as in control Vero cells and that the addition of exogenous Se does not alter the kinetics of virus replication.

However, it is interesting to note the increased severity of WNV-induced cytotoxicity and apoptosis in Se-deficient Vero cells. WNV infection is lethal to host cells and initiates caspase-dependent apoptotic cell death within 32 hr of infection [24,41,42]. The imbalance in the ratio of relative expression of apoptosis-inducing genes and caspase activation is a tightly controlled process that is subject to redox regulation [47]. Flaviviruses, such as dengue and JEV have been shown to induce activation of apoptotic signaling pathway mediated by ROS [48,49]. On the other hand, Se also alters cell survival genes, such as Bcl2 [50,51] and induces caspase activation [52]. These observations might explain our results of increased caspase activity in WNV-infected Se-deficient cells. WNV-infected cells are vulnerable to cell death due to activation of the caspase-signaling pathway [41,42], and Se deficiency further enhances the severity of apoptosis by further increasing the ROS, caspase activity and down regulating anti-apoptosis genes. Moreover, though the direct effect of Se deficiency on ΔΨm has not been demonstrated, it has been established that cell death by oxidative damage includes loss of ΔΨm and release of cytochrome c [53]. *In vitro *studies also validate that the presence of selenite can block both, loss in ΔΨm and cell death induced by H_2_O_2 _[54]. Our data for the first time reveals that WNV infection results in loss of ΔΨm, and that it is more severe in WNV-infected Se-deficient cells. Mitochondrial dysfunction is one of the early events in apoptosis by mitochondria-mediated caspase activation pathway in several virus infections [24,48]. Our data clearly demonstrates that the increased cytopathogenicity observed in Se deficient cells is mediated by caspase activation and disruption of mitochondrial function.

The increase in the LDH activity as observed in infected Vero cells grown in control media (Fig 5D) for 48 hr concurs with the previous data which demonstrated that WNV infection at low MOI (≤ 1) induces LDH release in Vero cells at 32 hr after infection when compared to infection with high MOI (≥ 10) where elevated LDH activity was observed at very early time points as a result of necrosis [24,48]. Similarly, decrease in cell viability of infected-control cells at the same time point confirms the cell death and apoptosis induced by WNV in Vero cells. However, the detrimental impacts of Se deficiency on WNV-infected cells were not known. The present study is the first to demonstrate the profound cytopathogenicity, increase in LDH activity and further decrease in cell viability in Se deficient cells upon WNV infection. Since this study is performed using an in vitro model, it also suggests that the CPE of Se deficiency are mainly due to impaired oxidative response, rather than impaired immune response. Similar effects were observed when vitamin C-deprived mice were infected with influenza virus [33]. There were no differences in the lung viral titer between vitamin C-adequate and -deficient mice but the lung pathology was much greater in vitamin C-deficient mice [33]. Jaspers and colleagues also reported that influenza virus-induced apoptosis and changes in cell morphology were greater in Se-deficient bronchial epithelial cells [19].

## Conclusion

Our data demonstrate that Se deficiency can be efficiently induced in Vero and SK-N-SH cells without significantly compromising cell growth and proliferation, and these cells can be used to study responses of WNV to the vital nutrient, Se. Though Se deficiency affects cell viability and enhances WNV-infection induced CPE, the WNV copy numbers *per se *do not differ suggesting that Se might be an important dietary nutrient for maintaining balance between cell death and cell survival genes by limiting OS in WNV infection. However, we did not address the effect of Se on WNV mutations and generation of quasispecies. Further studies are warranted to examine the role of Se deficiency-induced ROS in enhancing WNV mutations and selection of quasispecies with heightened virulence as demonstrated for CVB3/0 and influenza viruses [7,55].

## Materials and methods

### Se-deficient cell-culture system

Vero (monkey kidney epithelial) and SK-N-SH (human neuroblastoma) cells, purchased from the American Tissue Culture Collection (ATCC, Manassas, VA), were maintained in M199 and minimum essential medium Eagle (MEME), respectively, supplemented with 10% fetal bovine serum (FBS) (ATCC), 100 μg/mL penicillin-streptomycin and 10 μg/mL gentamicin (Gibco-BRL, Carlsbad, CA). To induce Se-deficient conditions, cells were grown in media supplemented with 1% FBS, 5 μg/mL insulin (Sigma, St. Louis, MO) and 0.5 μg/mL transferrin (Sigma). Se-adequate cells were concurrently cultured in the same media supplemented with 50 nM sodium selenite (Sigma). Se-deficient cells were maintained for 10 days, passaged every four days using serum-free trypsin-EDTA solution (TrypLE select, Gibco-BRL), and the media was changed every two or three days.

### Growth curves of Se-deficient and Se-adequate cells

Vero and SK-N-SH cells (3 × 10^4 ^cells/well) were seeded in 96-well plates and grown in control (10% FBS), Se-deficient and Se-adequate media for five days. Every 24 hr the cells were trypsinized, resuspended in 100 μL of media and counted using a cell viability analyzer (Vi-cell, Beckman Coulter, Fullerton, CA). All experiments were performed two times in triplicate.

### Measurement of cellular glutathione peroxidase (cGPX)

Vero and SK-N-SH cells (2.5 × 10^5 ^cells) were plated in T25 flasks and grown in control, Se-deficient and Se-adequate media and were passaged every 4 days. At days 3, 7 and 10, the cells were washed twice with PBS, lifted using a cell scraper into a chilled Eppendorf tube, and homogenized on ice in 200 μL of ice-cold buffer consisting of 50 mM Tris pH 7.5, 5 mM EDTA and 0.5 mM DTT. Lysates were clarified by centrifugation at 11,000 rpm for 10 min at 4°C and total protein concentrations were assayed using the Bradford Protein Assay (Bio-Rad Laboratories, Hercules, CA). 200 μg of protein was used to determine cGPX activity using the cellular GPx1 assay kit, according to the manufacturer's instructions (Calbiochem, EMD Biochemicals, San Diego, CA).

### Western blot analysis of antioxidant enzymes

Total cellular protein extracts were prepared from Vero and SK-N-SH cells grown in control, Se-deficient and Se-adequate media at days 3, 7 and 10 post-induction of Se deficiency. 40–60 μg of total cellular protein extract was fractionated on a 4–12% gradient SDS polyacrylamide gel, and then transferred onto 0.2 μm nitrocellulose filters (Bio-Rad Laboratories) as described previously [56]. Non-specific binding sites were blocked with 5% skim milk in 1× PBS with 0.1% Tween (PBST), and membranes were incubated overnight at 4°C with antibodies against GPx1, copper-zinc superoxide dismutase (CuZnSOD), manganese superoxide dismutase (MnSOD), inducible nitric oxide synthase (iNOS) (Calbiochem), catalase, (Cortex Biochem, San Leandro, CA) and β-actin (Sigma). After three vigorous washings with PBST, the membranes were further incubated with alkaline phosphatase (AP)-conjugated secondary antibodies for 2 hr at room temperature and developed using AP-conjugated substrate color development kit (Bio-Rad Laboratories).

### Cellular RNA extraction and RT-PCR analysis

Control, Se-deficient and Se-adequate Vero and SK-N-SH cells at days 3, 7 and 10 post-induction of Se deficiency were washed twice with 1× PBS and total cellular RNA was extracted and cDNA synthesized from 1 μg of RNA as described previously [56]. The mRNA transcripts of MnSOD were amplified and quantitated in the Bio-Rad iCycler iQ™ Multicolor Real-Time PCR Detection System using 3 μL of 1:10 diluted template, Bio-Rad 2× iQ™ SYBER^® ^Green supermix and 10 pmol each of forward (5'-TTCAATGGTGGTGGTCAT ATC-3') and reverse (5'-AACCTCAGCCTTGGACAC-3') primers, in a final reaction volume of 20 μL. β-actin gene was amplified using forward (5'-TCAGCAAGCAGGAGTATGACG-3') and reverse (5'-ACGCAACTAAGTCATAGTCCGC-3') primers and was used as an internal baseline reference. Thermal cycling was initiated with a first denaturation step of 4 min at 95°C, followed by 38 cycles of 95°C for 10 s and 56°C for 30 s, and the amplification fluorescence was read at 56°C. A standard curve for the PCR efficiency was constructed using serial dilutions of cDNA of control Vero and SK-N-SH cells starting at 50 ng and decreasing by 5-fold. All experiments were performed at least three times in duplicate and the data were analyzed for fold-change as described previously [56].

### Measurement of OS

At day 6 post-induction of Se deficiency, control, Se-deficient and Se- adequate Vero and SK-N-SH cells were plated in 96-well plates (4 × 10^4 ^cells/well) and the total ROS after 24 hr was measured, using the ROS-sensitive fluorescent 2',7' dichlorodihydrofluorescein diacetate (H_2_DCF-DA) probe (Invitrogen, Carlsbad, CA). After washing once with PBS, the cells were incubated with H_2_DCF-DA probe at the final concentration of 5 nM for 30 min at 37°C. After incubation, the cells were washed twice with PBS, resuspended in 200 μL of PBS and the fluorescence was read at 485/535 nm using a multiplate reader (Victor3, Perkin Elmer, MA).

### WNV infection and qRT-PCR for WNV copy number

A stock of lineage I WNV strain NY99 (1 × 10^9.7 ^PFU/mL), originally isolated from a crow in New York and propagated in Vero cells, was diluted to appropriate concentrations for infection experiments. Vero cells were seeded in either 6-well, coverslips in 24-well plates or 96-well plates to 80% confluency and inoculated at MOI 1 and adsorbed for 1 hr at 37°C. After incubation, unadsorbed virus was removed by washing twice with PBS and cells were incubated with the respective growth media: control (10% FBS), Se-deficient and Se-adequate. CPE was observed for 4 days after infection. Supernatants from 6-well plates were harvested every day and replaced with fresh medium until day 6 after infection for conducting viral copy number assays. Viral RNA from the supernatant was extracted using Qiaprep viral RNA extraction kit (Qiagen, Valencia, CA) and cDNA was synthesized using 10 μL of viral RNA as described above. WNV copy number was quantitated by using 2 μL of 1:10 diluted cDNA template and 12.5 pmol/μL each of forward (5'-ACAAGTCACCCTCACCGTTACG-3') and reverse (5'-GCCATCCACTACAGCGTTCTTC-3') primers specific for WNV NY99 NS4B gene as described above. Thermal cycling was initiated with a first denaturation step of 4 min at 95°C followed by 40 cycles of 95°C for 20 s and 57°C for 60 s, and the amplification fluorescence was read at 57°C. A standard curve with the dynamic range of detection in the range10^8 ^to 10^2 ^copies was constructed by preparing 10-fold serial dilutions of linear WNV NS4B gene. All experiments were performed at least three times in duplicate.

### Immunofluorescent antibody staining of WNV antigen

At day 2 after infection, WNV-infected (MOI1), mock-infected control, Se-deficient and Se-adequate Vero cells on coverslips were fixed in 4% paraformaldehyde for 10 min at room temperature, washed twice in 1× PBS and permeabilized in 0.5% TritonX 100 for 15 min. The coverslips were blocked with 4% BS in 1× PBS for 1 hr, washed three times in 0.1% BSA and incubated first with monoclonal human anti-WNV envelope antibody (1:800) at 4°C overnight and then with Alexa Fluor 488 conjugated goat anti-mouse secondary antibody (1:1000, Invitrogen). After washing with 0.1% BSA in 1× PBS, the cell nuclei were counterstained with bisbenzidine (1 ng/mL) (Cat#H33258, Sigma) before mounting with Vectashield mounting medium (Vector Laboratories, Burlingame, CA). Fluorescent cells were examined using a Zeiss Axiovert 200 microscope, equipped with appropriate fluorescence filters and objectives.

### Cytotoxicity and apoptosis assays

Cell viability of Vero cells grown in control, Se-deficient and Se-adequate media, was assessed prior to WNV infection, i.e., at day 7 post-induction of Se deficiency and then at day 2 after infection with WNV using CellTiter 96 AQ_ueous _One Solution Cell Proliferation Assay (Promega) as described previously [57]. Further for the analysis of WNV-induced cytotoxicity, the release of lactate dehydrogenase (LDH) was detected in infected and mock-infected control, Se-deficient and Se- adequate cells at day 2 after infection using Cytox-One assay kit (Promega) in accordance with the manufacturer's procedure. In brief, the LDH released by the cells in the supernatant was measured by reading the conversion of fluorescent compound resozurin to resorufin at excitation/emission wavelength of 560/590 nm. The concentration of LDH was expressed as fold-change in LDH activity in WNV-infected cells with respect to their respective mock-infected cells. Apoptosis in WNV-infected and mock-infected Vero cells grown in control, Se-deficient and Se-adequate media at day 2 after infection was determined using the Caspase-Glo™ 3/7 Assay kit (Promega) as per the manufacturer's protocol. The cells were grown and infected in 96-well white optiplate, incubated with 100 μL of caspase substrate in dark for 2 hr at room temperature and the luminescence at the end of the incubation period was measured using Perkin Elmer multiplate reader (Wallace? Victor^3^, Perkin Elmer Life Sciences).

### Measurement of mitochondrial membrane potential (ΔΨm)

ΔΨm was measured using fluorescent probe JC-1 (5,5',6,6'-tetrachloro-1,1',3,3'-tetraethylbenzimidazole carbocyanide iodide) (Molecular Probes, Eugene, OR) as described previously [58]. Briefly, WNV-infected and mock-infected Vero cells grown in 96-well plates, at day 2 after infection were washed once with PBS, and incubated with 5 μM/mL JC-1 for 20 min in dark at 37°C. After one wash with 1× PBS, 100 μL of PBS was added in each well and the red fluorescence was read at λ_em _= 590 nm and green fluorescence was read at λ_em _= 535 nm. The ratio of 590/535 nm was considered as the relative ΔΨm value. Valinomycin at final concentration of 100 μM (Molecular Probes) was used as positive control.

## List of abbreviations used

ΔΨ:, mitochondrial membrane potential; AP: Alkaline phosphatase; CPE: cytopathogenic effects; cGPX: Glutathione peroxidase; CuZnSOD: Copper-zinc superoxide dismutase; CVB3/0: Coxsackie virus B3; FBS: Fetal bovine serum; GPx1: Glutathione peroxidase; H_2_DCF-DA: 2',7' dichlorodihydrofluorescein diacetate; H3N2: Influenza A virus; Huh: human hepatoma; iNOS: inducible nitric oxide synthase; LDH: lactate dehydrogenase; MEME; minimum essential medium eagle; MOI: Multiplicity of infection; MnSOD: Manganese superoxide dismutase; OS: oxidative stress; PBST: 1× PBS with 0.1% Tween; RAW 264.7: mouse monocyte-macrophage cells; ROS: reactive oxygen species; Se: Selenium; SK-N-SH: human neuroblastoma cells; WNV: West Nile virus.

## Competing interests

The authors declare that they have no competing interests.

## Authors' contributions

Conception of the study (VRN and RY); design of the study (SV and VRN); development of the Se-deficient model (SV, YM, and VRN); Western blots (SV and CN); WNV infections (BC and YL); Manuscript draft preparation (SV and VRN) and editing (SV, VRN and RY). All authors read and approved the final manuscript.
